# Patterns and Treatment Strategies of Osimertinib Resistance in T790M-Positive Non-Small Cell Lung Cancer: A Pooled Analysis

**DOI:** 10.3389/fonc.2021.600844

**Published:** 2021-03-02

**Authors:** Chunsheng Wang, Kewei Zhao, Shanliang Hu, Minghuan Li, Yipeng Song

**Affiliations:** ^1^Department of Radiation, Yantai Yuhuangding Hospital, Yantai, China; ^2^Department of Radiation Oncology, Shandong Cancer Hospital and Institute, Shandong University, Jinan, China

**Keywords:** non-small cell lung cancer (NSCLC), epidermal growth factor receptor (EGFR), osimertinib, resistance patterns, treatment strategies

## Abstract

**Introduction:**

Osimertinib resistance is inevitable. The purpose of this study was to explore the predictive value of pretreatment clinical characteristics in T790M-positive non-small cell lung cancer NSCLC patients for the resistance pattern of osimertinib during tumor progression as well as the treatment strategy.

**Methods:**

We performed a literature search in the NCBI PubMed database to identify relevant articles and completed a pooled analysis based on 29 related published studies. The relationship between clinical characteristics, EGFR mutation type, previous treatment history and the gene mutation pattern at resistance to osimertinib was analyzed.

**Results:**

A total of 38 patients were included in the pooled analysis. Patients with an initial epidermal growth factor receptor EGFR mutation status of 19 deletions were more likely to have T790M loss (HR: 12.187, 95% CI: 2.186–67.945, p = 0.004). Patients with an initial EGFR mutation of L858R were more likely to have C797S mutations (HR: 0.063, 95% CI: 0.011–0.377, p = 0.002). The other factors (age, gender, ethnicity, smoking history, previous EGFR-TKI targeted therapy history, history of radiotherapy and chemotherapy) were not associated with the resistance pattern of osimertinib (all p > 0.05).

**Conclusions:**

The type of EFGR mutation in T790M-positive NSCLC patients prior to treatment can predict the resistance pattern to osimertinib. This finding plays a vital role and theoretical basis in guiding clinicians to formulate treatment strategies at the early stage of treatment and rationally combine drugs to overcome EGFR-TKI resistance.

## Introduction

Epidermal growth factor receptor tyrosine kinase inhibitors (EGFR-TKIs) are effective measures for the treatment of non-small cell lung cancer (NSCLC) patients with EGFR mutations. The EGFR T790M mutation is the main mechanism of first/second-generation EGFR-TKI resistance (1, 2). To overcome this mutation, third-generation EGFR-TKIs were developed. Osimertinib, also known as Tagrisso^®^ or AZD9291, is the first third-generation EGFR-TKI and was developed to selectively inhibit EGFR T790M- and EGFR-sensitive mutations (19 exon deletion mutations and 21 exon L858R point mutations). AURA2 and AURA extension phase II clinical trials showed that in patients with EGFR T790M mutations after the first generation of gefitinib and erlotinib, the objective response rate (ORR) of treatment with osimertinib is 62–70% (3, 4). The AURA3 phase 3 trial showed that compared with dual-agent chemotherapy, the administration of osimertinib can significantly improve the patient’s progression-free survival (PFS) (10.1 *vs* 4.4 months) and ORR (71 *vs* 31%) (5). More recently, the FLAURA trial showed that for untreated NSCLC patients with advanced EGFR mutations, compared with standard first-line EGFR-TKI therapy (gefitinib and erlotinib), osimertinib showed a significant advantage in PFS (18.9 *vs* 10.2 month). The overall survival (OS) of first-line treatment with osimertinib was 38.6 months, which was nearly 7 months longer than that of standard EGFR-TKI treatment (6, 7). Based on the AURA and the FLAURA study, osimertinib has been approved for second-line treatment of patients with T790M mutation positivity after EGFR-TKI treatment and first-line treatment of patients with EGFR-sensitive mutations. With the wide application of osimertinib as a first- and second-line therapy, its drug resistance problem is becoming increasingly obvious. The resistance of osimertinib has become a major obstacle to the treatment of EGFR-mutated NSCLC. The mechanisms of osimertinib resistance and treatment strategies have become the focus of attention.

The purpose of this pooled analysis was to investigate the predictive value of pretreatment clinical characteristics on the resistance pattern of osimertinib at tumor progression, as well as the treatment strategies for osimertinib resistance.

## Materials and Methods

### Search Strategy

We performed a literature search in NCBI PubMed database to identify all the relevant articles without language restriction (the last search update was January 31, 2020). The following search strategy were used:(((((osimertinib[Title/Abstract]) OR AZD9291[Title/Abstract])) AND ((epidermal growth factor receptor[Title/Abstract]) OR EGFR[Title/Abstract])) AND resistance[Title/Abstract]) AND ((non-small cell lung cancer [Title/Abstract]) OR NSCLC [Title/Abstract]).We also manually checked the references lists of all related articles to supplement more research.

### Study Eligibility and Data Extraction

Two authors (CS W and KW Z) independently screened the titles and abstracts of the search results and a second screening of the full-text articles. If these two authors failed to reach a consensus, a third investigator (SL H) was consulted to resolve the disagreements and reach a consensus on all items. Articles were included if they met the following inclusion criteria: 1) articles focusing on patients with non-small cell lung cancer; 2) prospective or retrospective studies, case reports and letters to the editor were all included due to the small number of relevant articles; 3) all patients harbored an initial EGFR mutation of L858R or 19 del and received first- or second-generation EGFR KTIs; 4) all patients underwent genetic testing before treatment with osimertinib and confirmed the presence of the T790M mutation; 5) all patients underwent the third genetic test after osimertinib failed; 6) treatment response to osimertinib, also known as complete response (CR), partial response (PR), stable disease (SD) or progressive disease (PD) were reported; 7) progression free survival (PFS) of osimertinib treatment were reported; 8) treatment response and PFS of pre-osimertinib EGFR TKIs were reported. For each eligible study, the following data were collected: age, gender, ethnicity, smoking history, tumor stage, initial mutation type, type of prior EGFR-TKIs, line of prior EGFR-TKIs, response to prior TKI, PFS of prior TKI, radiotherapy or chemotherapy history, EGFR mutation before and after osimertinib, EGFR mutation of prior EGFR mutation of osimertinib.

### Statistical Analysis

Fisher’s exact or chi-square tests were used to assess the associations between clinicopathological parameters and resistance pattern. Fisher’s exact or chi-square tests were used to assess the associations between clinicopathological parameters and treatment response to osimertinib. The Kaplan–Meier method and the log-rank test were used to analyze the association of clinicopathological parameters with PFS, and the associated 95% CIs were calculated. The analyses were performed with SPSS 22.0 program (SPSS Inc, Chicago, IL, USA), a two-sided p-value less than 0.05 was considered statistically significant.

## Results

### Search Results

**Figure 1:**
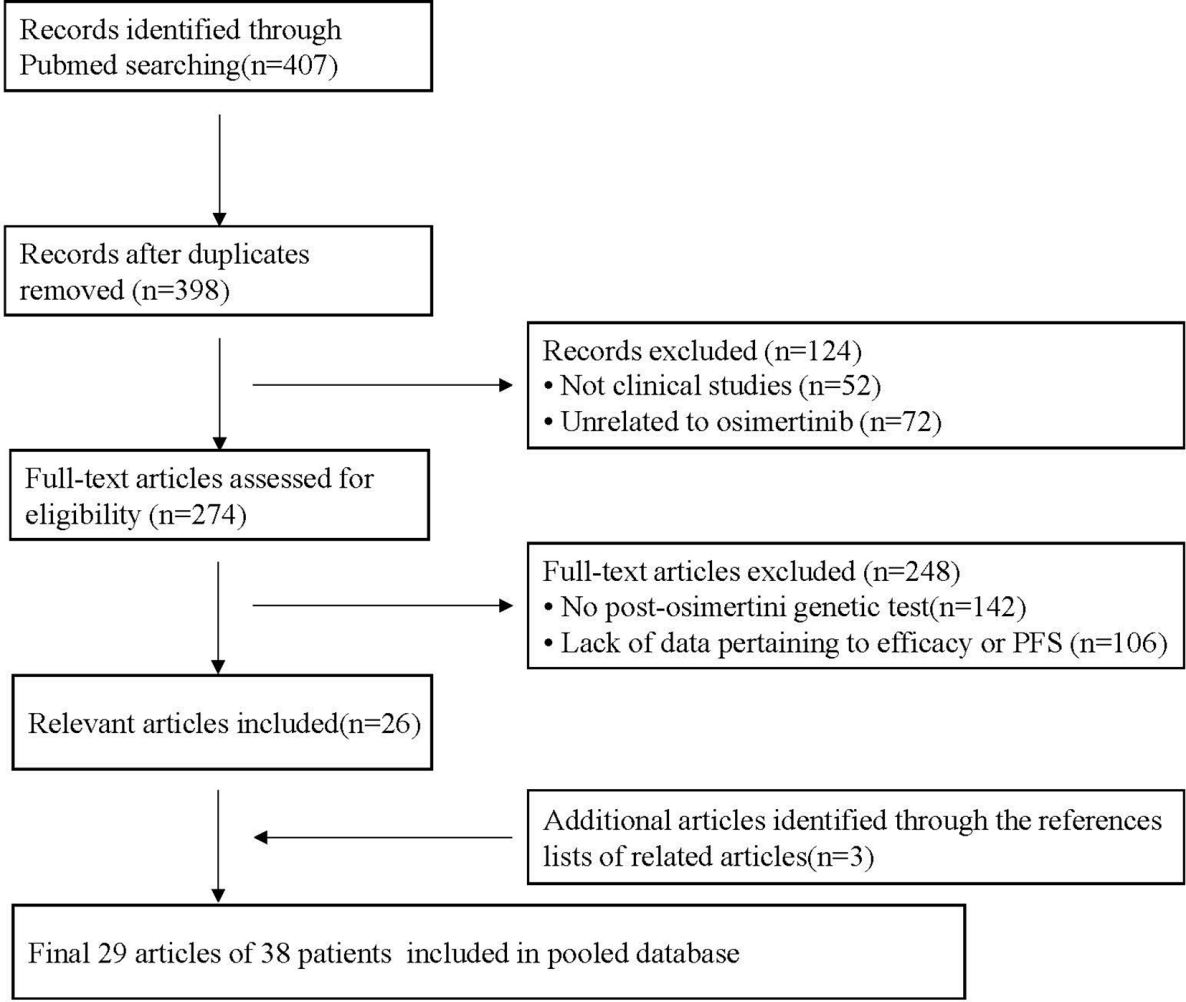
The flow chart of the study selection process.

A total of 407 potentially relevant articles were identified from the PubMed database. After removing duplicate records, 398 records remained. After preliminary evaluation by reading the title and abstract, 124 articles were excluded, including 52 non-clinical studies and 72 articles not related to osimertinib. The remaining 274 articles were further reviewed by reading the full text. Among them, 248 articles were excluded because 142 articles did not have genetic testing data after osimertinib resistance, and 106 articles lacked data pertaining to efficacy or PFS. Finally, 26 articles met the inclusion criteria, and another three articles were identified by retrieving the reference lists of these full-text evaluation articles. Overall, 29 articles were included in this pooled analysis. They were published between 2015 and 2019 and include one research article, five brief reports, four letters or communications and 19 case reports/case series (**Supplementary Table 1**). The flow chart of the study selection process is shown in **Figure 1**.

### Patient Characteristics

A total of 38 patients were included in the pooled analysis, with a median age of 56 years old, from 36 to 87 years old. Most patients were female (60.5% for women; 39.5% for men), including 16 Asian patients, accounting for 42.1%, and 22 non-Asian patients, accounting for 57.9%. Twenty-three patients harbored an initial gene mutation of 19 del, accounting for 60.5%, and 15 patients harbored the L858R mutation, accounting for 39.5%. All patients had a history of previous EGFR-TKI therapy, of which 20 received erlotinib (52.6%), 11 received gefitinib (28.9%), and seven received other types of EGFR-TKIs (7%), such as afatinib and icotinib. More than one-third of patients receive previous EGFR-TKIs as first-line therapy (27,71.1%). After previous treatment with EGFR-TKIs, 31 patients achieved objective tumor response, with an objective response rate of 81.6% and a median PFS of 12.1 months (95%CI:9.2–14.8). The baseline characteristics of the patients are detailed in **Table 1**.

**Table 1:** Baseline characteristics.

Characteristics	No. of patients (n = 38)	percentage
Age		
median (range)	56	36–87
Gender		
Male	15	39.5
Female	23	60.5
Ethnicity		
Asian	16	42.1
Non-Asian	22	57.9
Smoking		
Yes	10	26.3
No	16	42.1
NE	12	31.6
EGFR driver mutation		
19 del	23	60.5
L858R	15	39.6
Type of prior EGFR TKIs		
Gefitinib	11	28.9
Erlotinib	20	52.6
Others	7	18.4
Line of prior EGFR TKIs		
1L	27	71.1
≥2 L	11	28.9
Response to prior TKI		
OR	31	81.6
Non-OR	7	18.4
PFS of prior TKI		
median (95% CI)	12.1	9.2–14.8
RT and CT before Osimertinib		
Yes	20	52.6
No	18	47.4
Response to Osimertinib		
OR	27	71.1
Non-OR	11	28.9
PFS of Osimertinib		
median (95% CI)	9.0	7.5–10.6

### Analysis of Tumor Response and Survival in the Treatment of Osimertinib

After treatment with osimertinib, 31 patients achieved objective tumor response, with an objective response rate of 71.1% and a median PFS of 9.0 months (95%CI:7.5–10.6) (**Table 1**). Fisher’s exact test and the chi-square test show that the type of EGFR mutation as well as age, gender, ethnicity, smoking history, previous EGFR-TKI targeted therapy history, history of radiotherapy and chemotherapy were not related to tumor response to osimertinib (all p > 0.05) (**Supplementary Table 2**). Moreover, the Kaplan–Meier curve shows that the above factors are also not related to PFS (all p > 0.05) (**Supplementary Table 3**).

### Pattern Analysis of Osimertinib Resistance

**Figure 2:**
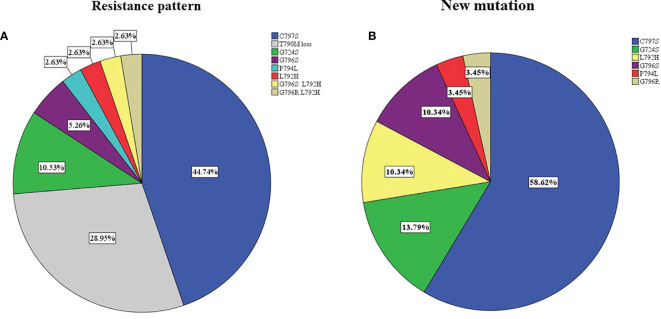
Resistance pattern **(A)** and new mutation site **(B)** analysis after osimertinib.

**Figure 3:**
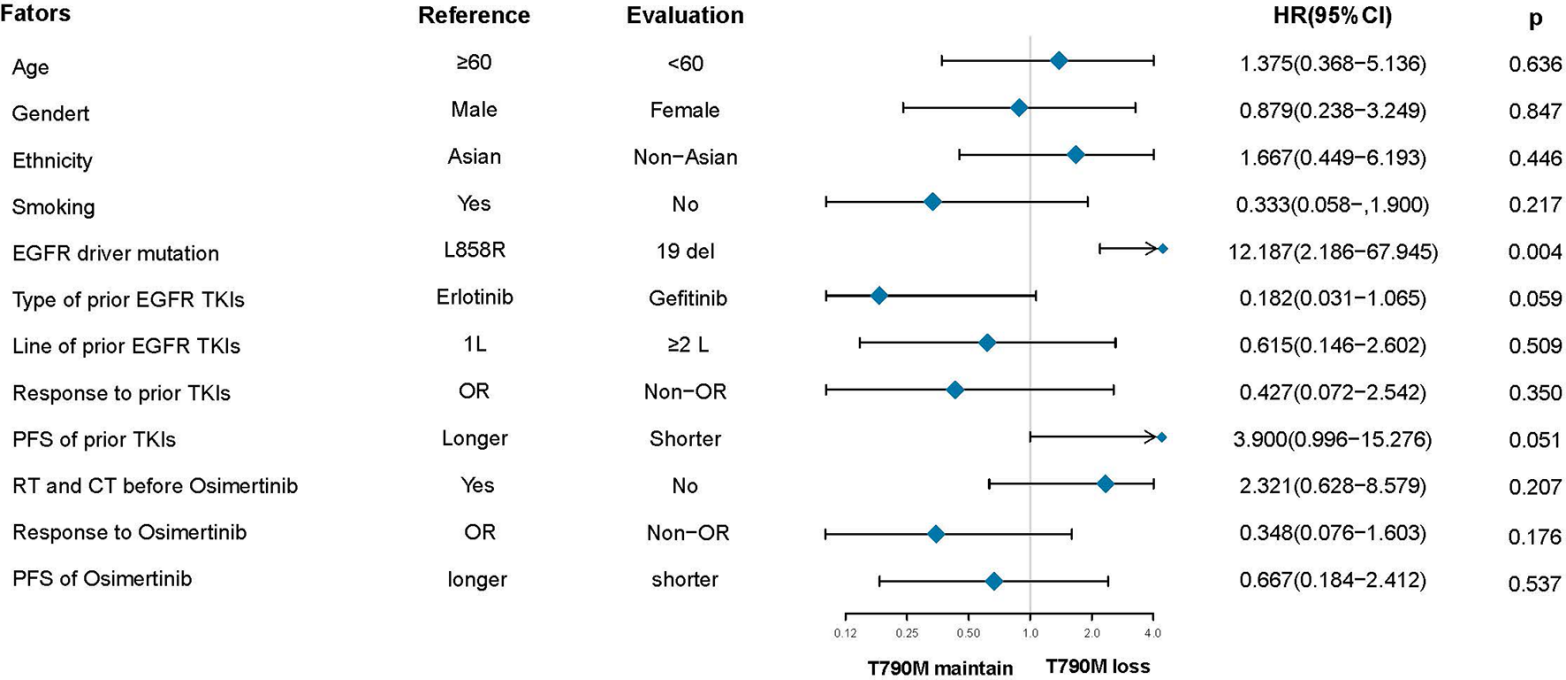
Subgroup analysis based on T790M status after resistance to osimertinib.

**Figure 4:**
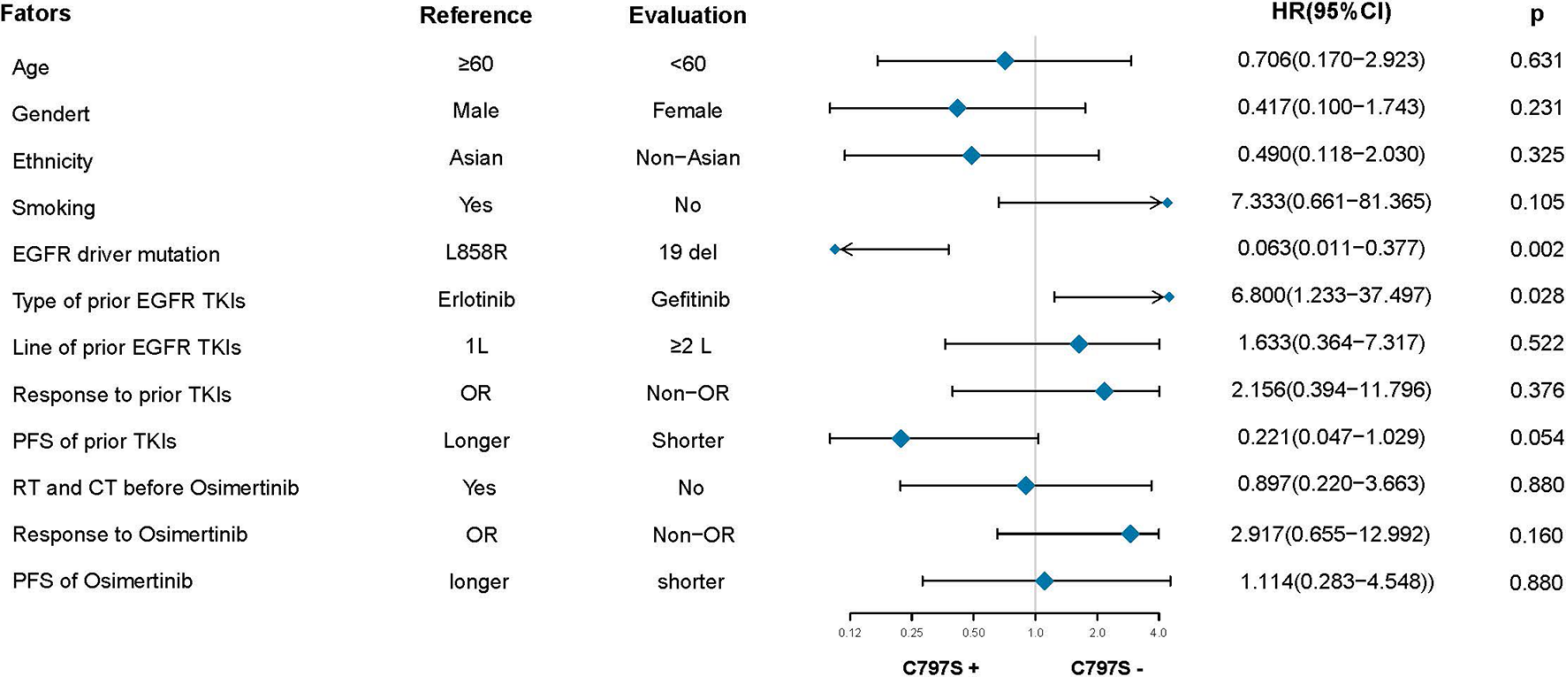
Subgroup analysis based on C797S status after resistance to osimertinib.

After the failure of osimertinib treatment, all patients underwent the third EGFR gene test. Eleven patients had T90M loss (28.95%), while other patients had new gene mutations. The new gene mutation patterns include C797S (17,28.95%), G724S (4,10.5%), G796S (2, 5.3%), G796R + L792H (1,2.6%), G796S + L792H (1,2.6%), L792H (1,2.6%) and P794L (1,2.6%), respectively (**Figure 2A**). From the perspective of a single gene, a total of 29 new gene mutations in six categories were found, namely, C797S (17,58.6%), G724S (4,13.8%), G796S (3,10.3%), L792H (3,10.3%), G796R (1,3.4%), P794L (1,3.4%), respectively (**Figure 2B**). According to the T790M status after the failure of osimertinib, the patients were divided into the T790M maintenance group and the T790M loss group, and the relationship between the clinical pathological characteristics of the two groups was compared. It was found that patients with an initial EGFR mutation status of 19 deletions were more likely to have T790M loss (HR:12.187,95%CI:2.186–67.945, p = 0.004). The other factors (age, gender, ethnicity, smoking history, previous EGFR-TKI targeted therapy history, history of radiotherapy and chemotherapy) were not significantly different between the two groups (all p > 0.05) (**Figure 3**). Next, the differences between the C797S-positive group and the C797S-negative group were compared according to the presence or absence of C797S. The results showed that patients with an initial EGFR mutation in L858R were more likely to have C797S mutations (HR: 0.063, 95% CI: 0.011–0.377, p = 0.002). Other factors between the two groups were not significantly different (all p> 0.05) (**Figure 4**).

## Discussion

The problem of EGFR-TKI resistance is inevitable. Similarly, the drug resistance mechanism and treatment of osimertinib urgently need to be solved in clinical practice. Our study found that the initial mutation type of EGFR is related to the resistance pattern of osimertinib in NSCLC patients. For patients with an initial mutation of 19 deletions, the drug resistance pattern is dominated by the loss of T790M, while for patients with the L858R mutation, the drug resistance pattern was mainly dominated by the generation of the third gene mutation C797S. This finding is of great significance for clinicians to formulate treatment strategies early to inhibit or overcome the emergence and development of drug resistance effectively and prolong the survival of NSCLC patients with EGFR mutations.

The C797S point mutation in exon 20 of EGFR is one of the most common mechanisms that mediates resistance to third-generation EGFR-TKIs (8–12). Thress et al. (11) first confirmed the presence of C797S in osimertinib-resistant patients with NSCLC. Peripheral blood specimen analysis of 19 patients with osimertinib resistance revealed that six of them had the C797S mutation (32%). In the AURA3 study with second-line osimertinib, genetic analysis of 73 patients with drug resistance showed that 10 (14%) of the patients had the C797S mutation, and all retained the T790M mutation (5). In the FLAURA study, among 91 patients who were resistant to osimertinib in the first line, the genetic test analysis showed that six patients had C797s mutations (7%) (6, 7). The researchers also confirmed the presence of C797S *in vitro* and found C797S mutations in cis and trans (C797S and T790M mutations on the same allele) and trans (two mutations on different alleles) (12). C797S-mediated resistance to osimertinib appears within 1 year of receiving osimertinib treatment (11). According to the different C797S mutation types, different treatment strategies need to be adopted. For the T790M/trans-C797S mutation, the combination of first- and third-generation EGFR-TKIs can be used to overcome the resistance of osimertinib (12–16). The first generation of EGFR-TKIs can inhibit the occurrence of the C797S mutation, while the third-generation EGFR-TKI osimertinib can effectively treat the EGFR T790M resistance mutation induced by first-generation EGFR-TKIs. Therefore, the combination of the first- and third-generation EGFR-TKIs may be an ideal therapeutic strategy to overcome the EGFR C797S/T790M osimertinib-resistant mutation occurring on different chromosomes. In vitro studies have reported that T790M/trans-C797S mutant cells are resistant to third-generation TKIs but are sensitive to the combined use of first- and third-generation EGFR TKIs (12, 13). Wang (14) reported the first complete clinical treatment case of erlotinib combined with osimertinib in the treatment of osimertinib-resistant patients with the 19del/T790M/trans-C797S mutation. A 42-year-old male patient was confirmed to have a T790M/C797S trans-mutation by genetic testing after osimertinib resistance, and then the patient received first- and third-generation EGFR-TKI combination therapy (erlotinib+osimertinib). The symptoms were significantly relieved within a week, and the PFS reached 3 months. Similarly, Zhou et al. reported a 42-year-old female patient with the osimertinib-resistant 19del/T790ertedM/trans-C797S mutation. After treatment with erotinib combined with osimertinib and bevacizumab, the patient maintained a stable disease with PFS lasting for 8 months (15). Based on the above research results, the researchers conducted a phase I/II clinical trial (NCT03122717) of osimertinib combined with gefitinib in the first-line treatment of EGFR-sensitive mutant advanced NSCLC. At the 2020 American Society of Clinical Oncology (ASCO), Julia (16) reported that the ORR of osimertinib combined with gefitinib was 85.2%, and the plasma EGFR mutation clearance rate of patients after 2 weeks of treatment was 82.4%. Osimertinib combined with gefitinib as a first-line treatment for EGFR-sensitive mutations in advanced NSCLC is tolerable, and it can quickly remove EGFR mutations in the plasma (16).Our research indicates that patients with an initial EGFR mutation of L858R are more likely to acquire resistance to osimertinib through the C797S mutation. In view of the above research results, the combination of gefitinib and osimertinib can be considered a first-line treatment for these patients to overcome the occurrence of the C797S mutation and avoid osimertinib resistance. For the T790M/cis-C797S mutation, there is currently no established treatment strategy for EGFR-TKIs alone or in combination (12, 13); however, brigatinib combined with cetuximab may play a key role (17–19). Brigatinib can overcome the triple mutation of EGFR/T790M/cis-C797S induced by osimertinib resistance by competitively affecting the activity of the EGFR kinase ATP domain. The binding of the EGFR antibody cetuximab to the EGFR receptor can internalize the EGFR receptor on the surface of the cell membrane and downregulate its expression. Brigatinib combined with the anti-EGFR antibody cetuximab can enhance the inhibition of EGFR phosphorylation and its downstream signaling pathways, significantly inhibiting tumor growth. The use of brigatinib alone or in combination with anti-EGFR antibodies can reduce tumor volume and prolong survival in mice with the 19del/T790M/C797S triple mutation (17). Wang X (18) reported the first effective clinical case of the treatment of 19del/T790M/cis-C797S triple mutation NSCLC with brigatinib combined with cetuximab. After receiving brigatinib and cetuximab, the patient’s symptoms improved significantly, tumor marker levels dropped significantly, and the patient maintained a stable disease with PFS reaching 9 months. A retrospective cohort study of 15 patients harboring triple mutations of EGFR 19del or L858R/T790M/cis-C797S showed that the objective response rate, disease control rate, and median PFS of patients receiving brigatinib combined with cetuximab were significantly better than those of patients receiving chemotherapy (ORR; 60 *vs* 10%; DCR: 100 *vs* 60%; mPFS: 14 *vs* 3/3.5 months) (19). In addition, the new target drugs and the fourth-generation EGFR-TKI also showed promising effects on the C797S mutation. Jia Y et al. reported the first EGFR tyrosine kinase allosteric inhibitor, EAI045, which, by binding to the allosteric site of the enzyme molecule, changes the conformation of the enzyme molecule, thereby inhibiting the enzymatic reaction. Combined with cetuximab, EAI045 induced significant tumor shrinkage in mouse models carrying the triple mutation L858R/T790M/C797S (10). To C (20) developed a small molecule allosteric inhibitor, JBJ-04-125-02, based on EAI045, which has improved the overall ability to inhibit EGFR mutations and has excellent inhibitory ability to C797S mutation. JBJ-04-125-02 does not depend on cetuximab; it can overcome C797S mutation resistance as a single agent compared to the previously known brigatinib + cetuximab. In addition, JBJ-04-125-02 and osimertinib can jointly bind to the mutant EGFR protein and exert a common inhibitory effect, thereby effectively inhibiting EGFR and its downstream signaling pathways, promoting tumor cell apoptosis, and effectively inhibiting tumor growth with a more obvious curative effect. At the 2019 ASCO, the updated data show that U3-1402, an antibody conjugated drug targeting HER-3, had a disease control rate of nearly 100% in patients with advanced NSCLC with EFGR mutations and was effective in patients with triple gene mutations L858R/T790M/C797S and 19 del/T790M/C797S (21). The American Association for Cancer Research (AACR) annual meeting in 2019 reported that the new generation of EGFR-TKIs, TQB3804, can not only inhibit the resistance of first-generation and second-generation EGFR-TKIs induced by the T790M mutation but also overcome the two common triple mutations, 19Del/T790M/C797S and L858R/T790M/C797S-, which occur after third-generation EGFR-TKI resistance (22). The 2019 ASCO reported the efficacy of the EGFR-cMET bispecific antibody JNJ-372 in NSCLC patients with different EGFR mutation subtypes, including the C797S mutation (23).

Loss of the T790M mutation was also found in patients with resistance to osimertinib. The AURA3 study showed that 49% (36/73) of patients had T790M loss after resistance to osimertinib (24). A retrospective analysis of the AURA study found that eight (44%) of the 18 patients who were resistant to osimertinib lost the T790M mutation (25). Moreover, A (26) and Oxnard GR et al. (27) reported that the frequency of T790M deletion in patients with resistance to osimertinib was as high as 68%. T790M loss was reported to be associated with early resistance to osimertinib and shorter survival benefits in patients (25–28). A recent study found that patients with T790M loss after osimertinib resistance had shorter mPFS than those with T790M mutation (29). Oxnard GR et al. (27) reported that the median time to treatment discontinuation of patients with T790M loss was lower than that of T790M maintenance patients. Zhao S et al. reported that patients with T790M loss progressed early to resistance to osimertinib, their median PFS was worse than those who maintained the T790M mutation, and patients in the T790M maintenance group had a relatively long survival benefit for osimertinib (28). Our study found that patients who maintained the T790M mutation had a slight advantage over patients who lost T790M in terms of treatment response and PFS. However, there was no statistical significance, which may be due to the relatively small sample size. T790M loss may be the result of osimertinib treatment, or it may be one of the causes of osimertinib resistance, which is related to tumor heterogeneity during tumor progression (27–31). Osimertinib can overcome the EGFR T790M mutation and may exert selective pressure, resulting in the increases in pre-existing T790M wild-type clones that carry additional EGFR-independent resistance mechanisms, making them more obvious than T790M mutant cells after osimertinib treatment (27, 28). However, the loss of T790M does not mean resensitization to the first generation of TKIs and often means overexpression of competitive mutation sites. A variety of EGFR-independent drug resistance mechanisms have been found in tumor cells with T790M loss, such as bypass signaling pathway activation (MET amplification, HER2 amplification, BRAF V600E mutation, PIK3CA mutation) and histological transformation (26–32). In contrast, for patients with T790M maintenance mutations at resistance to osimertinib, the original T790M mutant clones still dominate and progress to resistance to osimertinib by acquiring additional mutations such as C797S (28, 32). Based on these results and the effectiveness of osimertinib in inhibiting T790M-positive subcloning, repeated testing of T790M mutation status during the treatment process is helpful for the study of osimertinib resistance mechanisms and subsequent treatment strategies (27–32). However, testing of the T790M mutation status after osimertinib resistance appears to be slightly lagging. In this study, we found that patients with an initial EGFR mutation of 19 del are more likely to have T790M loss at resistance to osimertinib. Considering this resistance pattern and the fact that T790M loss is associated with early resistance to osimertinib, osimertinib combined with alternative pathway inhibitors (*e.g.*, MET inhibitors) may be considered in the case of early resistance. For patients with late-stage drug resistance, it may be appropriate to study osimertinib plus additional EGFR inhibitors, since the retention of EGFR-dependent drug resistance may be suspected (27).

We acknowledge that our research has some limitations. First, this is a pooled analysis of the published literature, most of which are included in case reports, and there may be some potential confounding factors beyond our control, such as publication bias and choice bias and residual confusion of unmeasured factors that cannot be ruled out. Second, the sample size of this study is relatively small, which may affect the accuracy of the results, and a large randomized controlled study is needed to verify our conclusions. Third, the genetic testing of the samples and methods adopted by patients may be different, which may affect the ability to detect gene mutations and the consistency of the results.

## Conclusions

In summary, this is the first study to report that the type of EFGR mutation in T790M-positive NSCLC patients prior to treatment can predict the resistance pattern to osimertinib. Patients with an initial EGFR mutation of L858R were more likely to have C797S mutations at resistance to osimertinib, while patients with an initial EGFR mutation status of 19 del were more likely to have T790M loss. This finding has important clinical significance, which plays a vital role and theoretical basis in guiding clinicians to formulate treatment strategies at the early stage of treatment and rationally combine drugs to overcome EGFR-TKI resistance.

## Data Availability Statement

The raw data supporting the conclusions of this article will be made available by the authors, without undue reservation.

## Author Contributions

Conceptualization, ML and YS. Methodology, ML and YS. Software, SH. Formal analysis, CW. Resources, KZ. Data curation, SH. Writing—original draft preparation, CW. Writing—review and editing, KZ. Supervision, ML and YS. Project administration, ML and YS. All authors contributed to the article and approved the submitted version.

## Conflict of Interest

The authors declare that the research was conducted in the absence of any commercial or financial relationships that could be construed as a potential conflict of interest.
