# Supplementary material for: Patterns and Treatment Strategies of Osimertinib Resistance in T790M-Positive Non-Small Cell Lung Cancer: A Pooled Analysis
Source: Front Oncol. 2021 Mar 2;11:600844. doi: 10.3389/fonc.2021.600844 (PMC7982860; doi:10.3389/fonc.2021.600844)
Supplement: Supplementary file 1 [file DataSheet_1.docx]

Supplement Table 1. The studies included for analysis

| Study | Number of articles | years | No. of patients | References* |
| --- | --- | --- | --- | --- |
| Research article | 1 | 2018 | 6 | ^1^ |
| Brief report | 5 | 2017-2019 | 5 | ^2-6^ |
| Letters or communication | 4 | 2017-2019 | 4 | ^7-10^ |
| Case reports/case series | 19 | 2015-2019 | 24 | ^11-29^ |

*: References here refer to the articles included in this pooled-analysis, not the references cited in the manuscript, which are listed in the reference section of the manuscript.

References

1. Goldberg ME, Montesion M, Young L, et al. Multiple configurations of EGFR exon 20 resistance mutations after first- and third-generation EGFR TKI treatment affect treatment options in NSCLC. *PLoS One.* 2018;13:e0208097.

2. Arulananda S, Do H, Musafer A, Mitchell P, Dobrovic A, John T. Combination Osimertinib and Gefitinib in C797S and T790M EGFR-Mutated Non-Small Cell Lung Cancer. *J Thorac Oncol.* 2017;12:1728-1732.

3. Castellano GM, Aisner J, Burley SK, et al. A Novel Acquired Exon 20 EGFR M766Q Mutation in Lung Adenocarcinoma Mediates Osimertinib Resistance but is Sensitive to Neratinib and Poziotinib. *J Thorac Oncol.* 2019;14:1982-1988.

4. Wang Z, Yang JJ, Huang J, et al. Lung Adenocarcinoma Harboring EGFR T790M and In Trans C797S Responds to Combination Therapy of First- and Third-Generation EGFR TKIs and Shifts Allelic Configuration at Resistance. *J Thorac Oncol.* 2017;12:1723-1727.

5. Zhang Q, Zhang XC, Yang JJ, et al. EGFR L792H and G796R: Two Novel Mutations Mediating Resistance to the Third-Generation EGFR Tyrosine Kinase Inhibitor Osimertinib. *J Thorac Oncol.* 2018;13:1415-1421.

6. Zhou Z, Zhao Y, Shen S, et al. Durable Clinical Response of Lung Adenocarcinoma Harboring EGFR 19Del/T790M/in trans-C797S to Combination Therapy of First- and Third-Generation EGFR Tyrosine Kinase Inhibitors. *J Thorac Oncol.* 2019;14:e157-e159.

7. Fairclough SR, Kiedrowski LA, Lin JJ, et al. Identification of osimertinib-resistant EGFR L792 mutations by cfDNA sequencing: oncogenic activity assessment and prevalence in large cfDNA cohort. *Exp Hematol Oncol.* 2019;8:24.

8. Fang W, Gan J, Huang Y, Zhou H, Zhang L. Acquired EGFR L718V Mutation and Loss of T790M-Mediated Resistance to Osimertinib in a Patient With NSCLC Who Responded to Afatinib. *J Thorac Oncol.* 2019;14:e274-e275.

9. Nie K, Jiang H, Zhang C, et al. Mutational Profiling of Non-Small-Cell Lung Cancer Resistant to Osimertinib Using Next-Generation Sequencing in Chinese Patients. *Biomed Res Int.* 2018;2018:9010353.

10. Oztan A, Fischer S, Schrock AB, et al. Emergence of EGFR G724S mutation in EGFR-mutant lung adenocarcinoma post progression on osimertinib. *Lung Cancer.* 2017;111:84-87.

11. Bersanelli M, Minari R, Bordi P, et al. L718Q Mutation as New Mechanism of Acquired Resistance to AZD9291 in EGFR-Mutated NSCLC. *J Thorac Oncol.* 2016;11:e121-123.

12. D P, Y L, F A, et al. EGFR-independent mechanisms of acquired resistance to AZD9291 in EGFR T790M-positive NSCLC patients. *Annals of Oncology.* 2015;26:2073-2078.

13. D Z, M H, Y B, et al. EGFR G796D mutation mediates resistance to osimertinib. *Oncotarget.* 2017;8:49671-49679.

14. Del Re M, Rofi E, Cappelli C, et al. The increase in activating EGFR mutation in plasma is an early biomarker to monitor response to osimertinib: a case report. *BMC Cancer.* 2019;19:410.

15. Fassunke J, Muller F, Keul M, et al. Overcoming EGFR(G724S)-mediated osimertinib resistance through unique binding characteristics of second-generation EGFR inhibitors. *Nat Commun.* 2018;9:4655.

16. Klempner SJ, Mehta P, Schrock AB, Ali SM, Ou SI. Cis-oriented solvent-front EGFR G796S mutation in tissue and ctDNA in a patient progressing on osimertinib: a case report and review of the literature. *Lung Cancer (Auckl).* 2017;8:241-247.

17. Knebel FH, Bettoni F, Shimada AK, et al. Sequential liquid biopsies reveal dynamic alterations of EGFR driver mutations and indicate EGFR amplification as a new mechanism of resistance to osimertinib in NSCLC. *Lung Cancer.* 2017;108:238-241.

18. Lee J, Shim JH, Park WY, et al. Rare Mechanism of Acquired Resistance to Osimertinib in Korean Patients with EGFR-mutated Non-small Cell Lung Cancer. *Cancer Res Treat.* 2019;51:408-412.

19. Liu J, Jin B, Su H, Qu X, Liu Y. Afatinib helped overcome subsequent resistance to osimertinib in a patient with NSCLC having leptomeningeal metastasis baring acquired EGFR L718Q mutation: a case report. *BMC Cancer.* 2019;19:702.

20. Liu Y, Li Y, Ou Q, et al. Acquired EGFR L718V mutation mediates resistance to osimertinib in non-small cell lung cancer but retains sensitivity to afatinib. *Lung Cancer.* 2018;118:1-5.

21. Ma L, Chen R, Wang F, et al. EGFR L718Q mutation occurs without T790M mutation in a lung adenocarcinoma patient with acquired resistance to osimertinib. *Ann Transl Med.* 2019;7:207.

22. Ou SI, Cui J, Schrock AB, et al. Emergence of novel and dominant acquired EGFR solvent-front mutations at Gly796 (G796S/R) together with C797S/R and L792F/H mutations in one EGFR (L858R/T790M) NSCLC patient who progressed on osimertinib. *Lung Cancer.* 2017;108:228-231.

23. Patro M, Gothi D, Vaidya S, Sah RB. A "triple whammy" in adenocarcinoma lung. *Lung India.* 2019;36:340-344.

24. Svaton M, Pesek M, Baxa J, Mukensnabl P, Benesova L, Minarik M. Patient with Three EGFR Mutations - Gradual Development of Resistance to Previous Targeted Treatment. *Klin Onkol.* 2017;31:53-58.

25. van Kempen LC, Wang H, Aguirre ML, et al. Afatinib in Osimertinib-Resistant EGFR ex19del/T790M/P794L Mutated NSCLC. *J Thorac Oncol.* 2018;13:e161-e163.

26. Wang J, Chen J. Positive response to Icotinib in metastatic lung adenocarcinoma with acquiring EGFR Leu792H mutation after AZD9291 treatment: a case report. *BMC Cancer.* 2019;19:131.

27. York ER, Varella-Garcia M, Bang TJ, Aisner DL, Camidge DR. Tolerable and Effective Combination of Full-Dose Crizotinib and Osimertinib Targeting MET Amplification Sequentially Emerging after T790M Positivity in EGFR-Mutant Non-Small Cell Lung Cancer. *J Thorac Oncol.* 2017;12:e85-e88.

28. Yu HA, Tian SK, Drilon AE, et al. Acquired Resistance of EGFR-Mutant Lung Cancer to a T790M-Specific EGFR Inhibitor: Emergence of a Third Mutation (C797S) in the EGFR Tyrosine Kinase Domain. *JAMA Oncol.* 2015;1:982-984.

29. Zhang Y, He B, Zhou D, Li M, Hu C. Newly emergent acquired EGFR exon 18 G724S mutation after resistance of a T790M specific EGFR inhibitor osimertinib in non-small-cell lung cancer: a case report. *Onco Targets Ther.* 2019;12:51-56.
