# Supplementary material for: Patterns and Treatment Strategies of Osimertinib Resistance in T790M-Positive Non-Small Cell Lung Cancer: A Pooled Analysis
Source: Front Oncol. 2021 Mar 2;11:600844. doi: 10.3389/fonc.2021.600844 (PMC7982860; doi:10.3389/fonc.2021.600844)
Supplement: Supplementary file 2 [file DataSheet_2.docx]

Supplement table 2 Analysis for treatment response of osimertinib treatment

| Characteristics | No. | OR | ORR | p |
| --- | --- | --- | --- | --- |
| Age |  |  |  |  |
| <60 | 23 | 16 | 69.6 | 0.802 |
| ≥60 | 15 | 11 | 73.3 |  |
| Gender |  |  |  |  |
| Male | 15 | 10 | 66.7 | 0.630 |
| Female | 23 | 17 | 73.9 |  |
| Ethnicity |  |  |  |  |
| Asian | 16 | 13 | 81.3 | 0.237 |
| Non-Asian | 22 | 14 | 63.6 |  |
| Smoking |  |  |  |  |
| Yes | 10 | 5 | 50.0 | 0.1.8 |
| No | 16 | 12 | 75.0 |  |
| NE | - | - | - |  |
| EGFR driver mutation |  |  |  |  |
| 19 del | 23 | 18 | 78.3 | 0.225 |
| L858R | 15 | 9 | 60.0 |  |
| Type of prior EGFR TKIs |  |  |  |  |
| Erlotinib | 20 | 15 | 72.7 | 0.662 |
| Gefitinib | 20 | 15 | 75.0 |  |
| Others | 7 | 4 | 57.1 |  |
| Line of prior EGFR TKIs |  |  |  |  |
| 1L | 27 | 21 | 77.8 | 0.152 |
| ≥2 L | 11 | 6 | 54.5 |  |
| Response to prior TKI |  |  |  |  |
| OR | 31 | 23 | 74.2 | 0.369 |
| Non-OR | 7 | 4 | 42.9 |  |
| PFS of prior TKI |  |  |  |  |
| longer | 18 | 12 | 66.7 | 0.572 |
| shorter | 20 | 15 | 75.0 |  |
| RT and CT before Osimertinib |  |  |  |  |
| Yes | 20 | 13 | 65.0 | 0.386 |
| No | 18 | 14 | 77.8 |  |
| EGFR mutation before Osimertinib |  |  |  |  |
| 19 del +T790M | 23 | 18 | 78.3 | 0.225 |
| L858R+T790M | 15 | 9 | 60.0 |  |

Abbreviations: SUV_TLR:_ tumor-to-liver SUVmax ratio; SUV_TBR_: tumor-to-blood pool SUVmax ratio.

Supplement table 3 Analysis for PFS of osimertinib treatment

| Variable | mPFS | HR | 95%CI |  |
| --- | --- | --- | --- | --- |
| Age |  |  |  |  |
| <60 | 8.2 | Ref. |  |  |
| ≥60 | 10.0 | 0.830 | 0.423-1.628 | 0.580 |
| Gender |  |  |  |  |
| Male | 9.5 | Ref. |  |  |
| Female | 9.0 | 1.009 | 0.522-1.951 | 0.978 |
| Ethnicity |  |  |  |  |
| Asian | 9.0 | Ref. |  |  |
| Non-Asian | 9.0 | 0.836 | 0.434-1.608 | 0.591 |
| Smoking |  |  |  |  |
| Yes | 7.0 | Ref. |  |  |
| No | 10.0 | 0.576 | 0.250-1.327 | 0.195 |
| NE |  | - | - | - |
| EGFR driver mutation |  |  |  |  |
| 19 del | 9.0 | Ref. |  |  |
| L858R | 9.5 | 1.340 | 0.682-2.632 | 0.396 |
| Type of prior EGFR TKIs |  |  |  |  |
| Erlotinib | 9.0 | Ref. |  |  |
| Gefitinib | 10.7 | 0.656 | 0.303-1.423 | 0.286 |
| Others | - | - | - | - |
| Line of prior EGFR TKIs |  |  |  |  |
| 1L | 10.0 | Ref. |  |  |
| ≥2 L | 9.0 | 0.593 | 0.282-1.246 | 0.168 |
| Response to prior TKI |  |  |  |  |
| OR | 9.5 | Ref. |  |  |
| Non-OR | 8.0 | 1.147 | 0.500-2.629 | 0.746 |
| PFS of prior TKI |  |  |  |  |
| longer | 9.0 | Ref. |  |  |
| shorter | 8.0 | 0.975 | 0.500-1.900 | 0.940 |
| RT and CT before Osimertinib |  |  |  |  |
| Yes | 11.0 | Ref. |  |  |
| No | 8.0 | 0.725 | 0.376-1.397 | 0.337 |
| EGFR mutation before Osimertinib |  |  |  |  |
| 19 del +T790M | 9.0 | Ref. |  |  |
| L858R+T790M | 9.5 | 1.147 | 0.500-2.629 | 0.746 |
| Response to Osimertinib |  |  |  |  |
| OR | 10.7 | Ref. |  |  |
| Non-OR | 7.0 | 3.173 | 1.440-6.987 | 0.004 |
